# Long-Term Clinical Outcomes of Minimally Invasive Direct Coronary Artery Bypass Grafting

**DOI:** 10.3390/jcm14217590

**Published:** 2025-10-26

**Authors:** Sleiman Sebastian Aboul-Hassan, Maria Luszczyn, Ryszard Stanislawski, Maciej Peksa, Marcin Nawotka, Siarhei Amelchanka, Lukasz Moskal, Tomasz Stankowski, Romuald Cichon

**Affiliations:** 1 Department of Cardiac Surgery, Zbigniew Religa Heart Center “Medinet”, 67-100 Nowa Sol, Poland; marialuszczyn1@gmail.com (M.L.); ryh70@o2.pl (R.S.); ampeksa@gmail.com (M.P.); mnawotka79@gmail.com (M.N.); omelchenkosergey2@gmail.com (S.A.); lk.moskal@gmail.com (L.M.); romuald.cichon@gmx.de (R.C.); 2 Department of Cardiac Surgery and Interventional Cardiology, Faculty of Medicine and Medical Sciences, University of Zielona Gora, 65-046 Zielona Gora, Poland; 3 The Student Society of Cardiac Surgery, Collegium Medicum University of Zielona Gora, 65-046 Zielona Gora, Poland; 4 Department of Cardiac Surgery, Sana Heart Center Cottbus, 03048 Cottbus, Germany; tomekstankowski89@gmail.com

**Keywords:** minimally invasive direct coronary artery bypass, MIDCAB, coronary artery bypass grafting, CABG, hybrid coronary revascularization, minimally invasive surgery, LITA–LAD graft, off-pump surgery

## Abstract

**Background/Objectives**: Minimally invasive direct coronary artery bypass (MIDCAB) surgery, performed through a left minithoracotomy, has emerged as an alternative to conventional coronary artery bypass grafting (CABG), which requires a full sternotomy. This procedure is ideal for patients with isolated proximal left anterior descending (LAD) artery disease or high surgical risk. The aim of this study was to assess the long-term clinical outcomes of MIDCAB performed at a single center with stratification by revascularization strategy. **Methods**: A total of 480 patients who underwent off-pump MIDCAB between 2012 and 2024 at a single center were retrospectively analyzed and categorized into three distinct groups: complete revascularization (MIDCAB-CR), hybrid coronary revascularization (MIDCAB-HCR) and incomplete revascularization (MIDCAB-IR). Short- and long-term outcomes, including mortality, major adverse cardiac and cerebral events (MACCE) and LITA–LAD graft patency were evaluated. Median follow-up was 3.39 years. **Results**: In-hospital mortality was 1.4%. At a median follow-up duration of 3.39 years, the overall LITA–LAD graft patency was 94.4% with 5- and 10-year survival rates of 78% and 60%, respectively. MIDCAB-CR and MIDCAB-HCR groups showed comparable long-term survival and freedom from MACCE, both significantly better than those observed in the MIDCAB-IR groups. **Conclusions**: These findings support the safety and durability of MIDCAB as an effective revascularization strategy, especially when performed as complete or hybrid revascularization. Incomplete revascularization may be considered in selected high-risk patients but is associated with worse outcomes.

## 1. Introduction

Coronary artery bypass grafting (CABG) is one of the most common procedures performed in adult cardiac surgery and it is the treatment of choice for patients with multivessel coronary artery disease [1,2]. During CABG, the use of left internal thoracic artery (LITA) to graft the left anterior descending (LAD) artery is considered the gold standard of care.

The development of minimally invasive direct coronary artery bypass grafting (MIDCAB) has been made possible by advances in medical technology [3]. MIDCAB offers several significant advantages over conventional CABG via sternotomy, resulting in a faster recovery time and an improved quality of life due to limited surgical injury [4,5,6]. Moreover, a recent study showed that MIDCAB LITA to LAD is associated with superior freedom from myocardial infarction (MI) and target vessel repeat revascularization compared with second-generation drug eluting stents [7].

Despite these advantages, the adoption rate of minimally invasive CABG in the surgical community is limited and does not exceed 1.5% of annual CABG volume, according to the most recent Society of Thoracic Surgeons database report [1]. At our institution, MIDCAB has been the preferred strategy since 2012, mainly in patients with isolated proximal LAD or in high-risk patients with multivessel disease as part of a hybrid revascularization strategy. Therefore, the aim of this study was to evaluate the long-term outcomes in a large series of patients who had undergone MIDCAB.

## 2. Materials and Methods

### 2.1. Study Design

**Figure 1:**
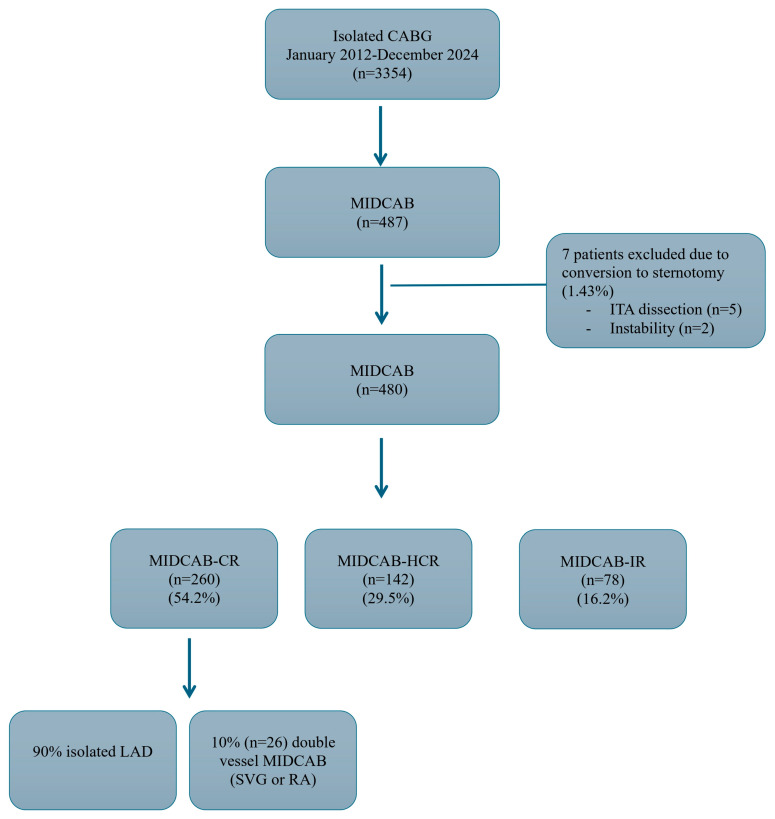
The flow diagram details the selection of patients within each group. The diagram presents a visual representation of the included and excluded patients. CABG: Coronary artery bypass grafting; MIDCAB: Minimally invasive direct coronary artery bypass; ITA: Internal thoracic artery; CR: Complete revascularization; HCR: Hybrid coronary revascularization; IR: Incomplete revascularization; LAD: Left anterior descending artery; SVG: Saphenous vein graft; RA: Radial artery.

Between January 2012 and December 2024, 487 patients underwent MIDCAB procedure through small left anterolateral minithoracotomy at Zbigniew Religa Heart Center “MEDINET”, Nowa Sol, Poland. We performed a retrospective analysis of prospectively collected data from the surgical database. The data were collected and reported in accordance with the Polish National Registry of Cardiac Surgery Procedures (KROK) (https://krok.csioz.gov.pl), a mandatory database for every cardiac surgery department in Poland. This database captures detailed preoperative, intraoperative, and postoperative hospital variables for all patients undergoing cardiac surgery. Additionally, we retrieved surgical protocols and discharge notes for each patient from the database. Of those 487 patients, 7 patients had conversion to sternotomy (1.43%) due to LITA dissection (*n* = 5) or hemodynamic instability (*n* = 2) and those patients were excluded from the final analysis. A total of 480 patients were enrolled in this study and assessed in 3 different groups according to their revascularization strategy (Figure 1 represents patients flow chart diagram):

- MIDCAB CR (CR-Complete revascularization) group (*n* = 260): 234 patients (90.0%) had isolated LAD disease and the rest (10.0%) had double vessel disease MIDCAB using either saphenous vein graft or radial artery connected to the LITA as a Y/T graft with LITA revascularizing the LAD.
- MIDCAB hybrid coronary revascularization (HCR) group (*n* = 142): these patients had multivessel disease with the heart team decision to perform percutaneous coronary intervention (PCI) and MIDCAB. 135 patients (95%) had isolated MIDCAB LITA to LAD and the rest (5%) had had double vessel disease MIDCAB using either saphenous vein graft or radial artery connected to the LITA as a Y/T graft with LITA revascularizing the LAD. First PCI strategy was performed in 61 patients (42.9%) and the rest 81 patients (57.1%) first MIDCAB strategy was performed.
- MIDCAB IR (IR-Incomplete revascularization) group (*n* = 78): these patients had multivessel disease with the heart team decision to perform planned IR with only MIDCAB LITA-LAD due to high-risk profile of the patients and unfavorable PCI anatomy (IR was defined as an untreated significant stenosis of more than 70% of the vessel diameter).

### 2.2. Surgical Technique

**Figure 2:**
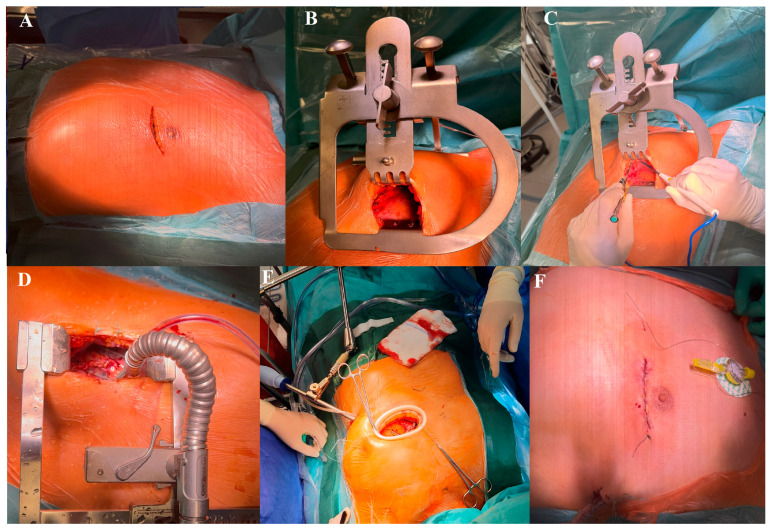
(**A**) A 6–7 cm anterolateral left minithoracotomy incision. (**B**) A specialized retractor used for left internal thoracic artery (LITA) harvest through a left anterior minithoracotomy. (**C**) Under direct vision, LITA was harvested in a skeletonized or pedicled fashion using low energy long blade cautery. (**D**,**E**) Different mechanical epicardial stabilizers used to perform off-pump LITA-LAD anastomosis. (**F**) The left anterior thoracotomy scar with a single left-sided pleural drain after completion of surgery.

Patients undergoing MIDCAB were intubated using a double-lumen endotracheal tube. Then, an inflatable bean bag was placed under the left hemithorax while the patient was in the supine position. A 6–7 cm anterolateral minithoracotomy incision was performed (Figure 2A) and the pleural space was entered usually through the 4th intercostal space with the left lung deflated. In the majority of the patients, LITA was harvested under direct vision using dedicated MIDCAB retractor. During the study period three MIDCAB retractors were used (CTS MIDCAB retractor, STORZ MIDCAB retractor and Delacroix-Chevalier MIDCAB retractor) (Figure 2B). Under direct vision, LITA was harvested in a pedicled or skeletonized fashion using low energy long blade cautery (Figure 2C). In 15 patients, LITA was harvested thoracoscopically using harmonic scalpel (Ethicon). Following heparinization, LITA-LAD anastomosis is performed off-pump with the aid of the mechanical epicardial stabilizer (Figure 2D,E). Coronary shunts were not used routinely. The activated clotting time was maintained above 400 s after systematic heparinization. Flow measurements were performed routinely in all patients for the past 3 years. During the study period, MIDCAB was performed by 5 trained surgeons (S.S.A.H, R.S, M.P, M.N, S.A)

### 2.3. Study Endpoints and Definitions

Early outcomes included: in-hospital mortality, myocardial infarction (MI), stroke, deep wound infection (DWI), reoperation due to bleeding, new onset renal replacement therapy (RRT), the use of Intra-aortic balloon pump (IABP), packed red blood cell transfusion (pRBC) and duration of hospital stay (HS). MI was defined according to the fourth international definition of MI [8]. Stroke was defined as the development of a new permanent neurologic deficit as confirmed by a neurologist, computed tomography, magnetic resonance imaging, or at autopsy examination. DWI was defined according to criteria established by the Center for Disease Control and Prevention [9,10].

The long-term outcomes were all-cause mortality, the incidence of major adverse cardiac and cerebral events (MACCE) (a composite endpoint including mortality, myocardial infarction, stroke, and repeat revascularization), and graft patency at follow-up. All-cause mortality was assessed, as it is considered an objective and unbiased endpoint for comparative studies [11]. Graft patency was evaluated using the Fitzgibbon classification system [12]. Patent grafts were defined as Fitzgibbon Class A (an excellent or unimpaired graft, or graft stenosis of less than 50%), while failed grafts were defined as Fitzgibbon Class B or O. Angiographies were assessed by an interventional cardiologist and/or a clinical cardiologist/radiologist.

We retrieved follow-up data from the National Health Care Registry of the Ministry of Health of the Republic of Poland, which stores and analyzes all health-related data. Once coronary angiography or cardiac computed tomography was noted in the registry, we contacted the local cardiology and radiology departments to obtain the angiographies. If a patient had more than one postoperative angiogram, only the latest (with the longest duration) was used, unless coronary intervention was performed or a failed graft was observed; in that case, the angiogram with intervention or the failed graft was used. Follow-up continued through February 2025.

Data on postoperative clinical outcomes were available for all patients in this study. Of the 131 patients who had one or more angiograms at follow-up registered in the national health database, we were able to retrieve angiograms for 109 patients (83.2%). This study was approved by the Institutional Review Board at University of Zielona Gora, Poland (RCM-CM-KBUZ.001.33.2025). Individual consent for anonymous data analysis was waived by the Committee.

### 2.4. Statistical Analysis

Continuous variables were expressed as mean and standard deviation (SD) or median and interquartile range (IQR), while categorical variables were expressed as number and percentage. Moreover, the normality of continuous variables was tested using the Shapiro–Wilk test. Student’s *t*-test was used for comparisons between groups of continuous data, while categorical variables were compared using the Pearson chi-squared test.

To reduce the impact of confounders inherent to observational studies, propensity score (PS) matching was applied to the three groups according to their revascularization strategy. Propensity scores were generated using a multivariable logistic regression model based on the preoperative variables listed in Table 1. 3 separate PS matched analysis were performed (MIDCAB-CR vs. MIDCAB-HCR, MIDCAB-CR vs. MIDCAB-IR and MIDCAB-HCR vs. MIDCAB-IR). In all three analyses, patients were matched in a 1:1 fashion using a caliper matching method without replacement and a caliper width of 0.2 standard deviations of the logit of the PS. The balance of the covariates was tested using the standardized mean difference (SMD). Due to the small sample size between groups, an SMD greater than 0.25 was considered a serious imbalance (supplementary data). The matched data were analyzed using matched-analysis procedures.

We estimated survival and freedom from MACCE using the Kaplan–Meier method. Hazard ratios were calculated using the Cox proportional hazards regression model. For the matched populations, the HRs were calculated using a stratified Cox proportional hazards regression analysis of matched pairs. The hazard ratios (HRs) and their corresponding 95% confidence intervals (95% CIs) are reported. A *p*-value of less than 0.05 was considered statistically significant in a two-tailed test.

All statistical analyses were performed using R Version 4.3.1 (R Core Team, 2021). R: A Language and Environment for Statistical Computing. R Foundation for Statistical Computing, Vienna, Austria. URL: https://www.R-project.org.

## 3. Results

**Figure 3:**
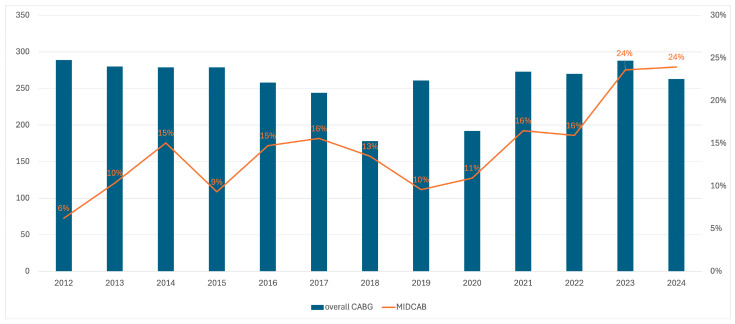
Bar-line chart representing the number of overall coronary artery bypass grafting (CABG) cases and the percentage of performed minimally invasive coronary artery bypass (MIDCAB) cases performed each year between 2012 and 2024.

Patient characteristics are shown in Table 1. The number of patients who underwent MIDCAB between 2012 and 2014 increased from 6% in 2012 to 23% in 2024 of the annual number of CABG cases per year (Figure 3). Of the initial 487 patients, 7 patients had conversion to sternotomy (1.43%) due to LITA dissection (*n* = 5) or hemodynamic instability (*n* = 2) and those patients were excluded from the final analysis. A total of 54.2% of the patients had MIDCAB-CR, 29.5% were in the MIDCAB-HCR group and 16.2% were in the MIDCAB-IR group. Median logistic European System for Cardiac Operative Risk Evaluation II (Euroscore II) in the whole cohort was 1.16% (interquartile range [IQR], 0.85–2.01). According to the revascularization strategy, patients in the MIDCAB-IR had worse risk profile (Euroscore II: 1.55%, IQR: 1.04–2.67) compared to MIDCAB-HCR (Euroscore II: 1.45%, IQR: 0.97–2.36) and MIDCAB-CR (Euroscore II: 0.99%, IQR: 0.78–1.62). Postoperatively, all patients received aspirin 6 to 24 h after surgery and continued to receive it daily thereafter. If recommended, the second antiplatelet agent was administered between 24 and 48 h after surgery. In the PCI group, all patients received dual antiplatelet therapy for at least 12 months. Other medications such as statins, beta-blockers and renin-angiotensin-aldosterone system inhibitors were used in 95.6%, 97.2% and 63.5% of patients, respectively. PS matching resulted in three well balanced matched pairs (103 pairs of MIDCAB-CR and MIDCAB-HCR, 55 pairs of MIDCAB-CR and MIDCAB-IR and 73 pairs of MIDCAB-HCR and MIDCAB-IR).

### 3.1. Early Outcomes

Early outcomes are presented in Table 2. All patients included in this study underwent off-pump MIDCAB. The median length of surgery was 119 min (IQR: 97–140). Seven patients (1.4%) died in the early postoperative period. Perioperative MI occurred in 20 patients (4.1%); of those, 14 patients had repeat revascularization. Since the routine adoption of intraoperative transient time flow measurement (TTFM) in 2022, the incidence of perioperative myocardial infarction (MI) has decreased significantly compared to the time prior to the routine use of TTFM (0.57% vs. 6.2%, *p* = 0.018). Early stroke occurred in 3 patients (0.6%). The overall incidence of early MACCE occurred in 27 patients (4.8%).

Surgical revision for bleeding was required in 29 patients (6%), which was resolved through the same thoracotomy. DWI occurred in 16 patients (3.3%). A transfusion of pRBC was performed on 107 patients (22.2%). The median length of hospital stay was 5.4 days (IQR 4.5–6.3).

According to the revascularization strategy, patients undergoing MIDCAB-IR had worse early outcomes, particularly with regard to early MACCE compared to patients with MIDCAB-CR and MIDCAB-HCR (Table 2).

### 3.2. Long-Term Outcomes

**Figure 4:**
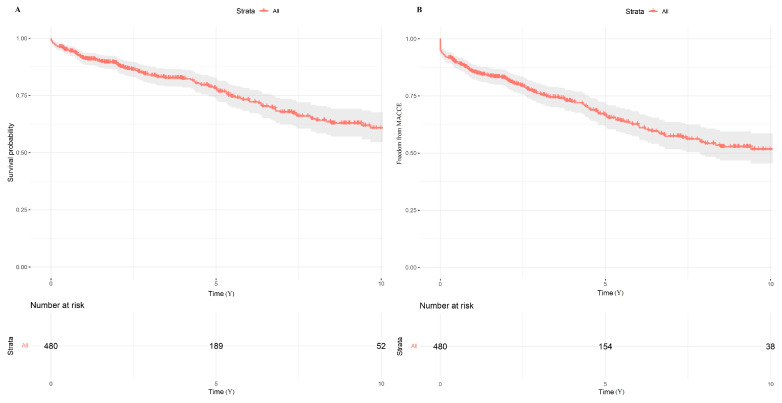
Kaplan–Meier curve probabilities in patients after minimally invasive direct coronary artery bypass grafting (MIDCAB). (**A**) Survival probability; (**B**) Freedom from major adverse cardiac and cerebral events (MACCE).

The median follow-up time for patients who underwent the MIDCAB procedure was 3.39 years (interquartile range [IQR]: 1.30–7.32 years). 5 and 10-year survival probabilities were 78% and 60%, respectively (Figure 4A). In addition, 5- and 10-year freedom from major adverse cardiac and cerebrovascular events (MACCE) were 66% and 51%, respectively (Figure 4B). Of the 131 patients who had one or more angiograms at follow-up registered in the national health database, we were able to retrieve angiograms for 109 patients (83.2%). The mean time from surgery to cardiac computer tomography scan or coronary angiogram was 1.44 years ± 2.15 years, and the LITA-LAD patency rate was 94.4%.

**Figure 5:**
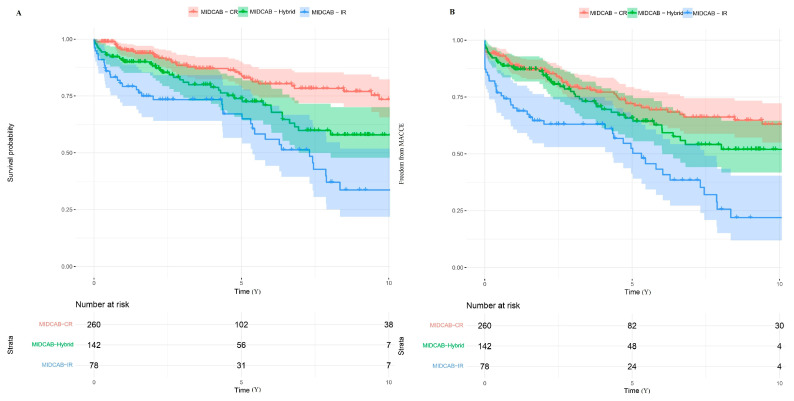
Kaplan–Meier curve probabilities in the minimally invasive direct coronary artery bypass (MIDCAB) cohort according to the revascularization strategy. (**A**) Survival probability; (**B**) Freedom from major adverse cardiac and cerebral events (MACCE). MIDCAB-CR—MIDCAB with completer revascularization. MIDCAB-IR—MIDCAB with incomplete revascularization.

Regarding the revascularization strategy, the 5- and 10-year survival probabilities in patients receiving MIDCAB-CR, MIDCAB-HCR, and MIDCAB-IR were 84.6% vs. 73.9% vs. 67.1% and 73.5% vs. 57.9% vs. 33.7%, respectively (Figure 5A). Freedom from MACCE at 5 and 10 years was 72.1% vs. 65.7% vs. 52.4% and 63.0% vs. 52.0% vs. 22.0%, respectively (Figure 5B). In the unmatched population, patients receiving MIDCAB-CR had improved survival (HR: 0.52; 95% CI [0.33–0.92]; *p* = 0.004), though freedom from MACCE was comparable (HR: 0.74; 95% CI [0.50–1.09]; *p* = 0.13) to that of patients receiving MIDCAB-HCR. Conversely, patients who received MIDCAB-CR or MIDCAB-HCR experienced improved survival (HR: 0.29; 95% CI [0.18–0.46]; *p* < 0.001) and (HR: 0.56; 95% CI [0.35–0.88]; *p* = 0.001), respectively, and had a higher freedom from MACCE than patients receiving MIDCAB-IR (HR: 0.37; 95% CI [0.25–0.55]; *p* < 0.001) and (HR: 0.50; 95% CI [0.33–0.76]; *p* = 0.001), respectively.

**Figure 6:**
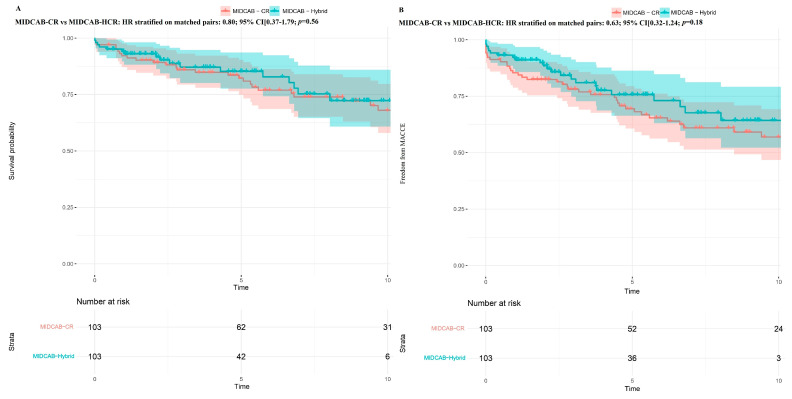
Kaplan–Meier curve probabilities in the matched cohort after minimally invasive direct coronary artery bypass (MIDCAB) with complete revascularization (CR) and hybrid revascularization strategy. (**A**) Survival probability; (**B**) Freedom from major adverse cardiac and cerebral events (MACCE).

**Figure 7:**
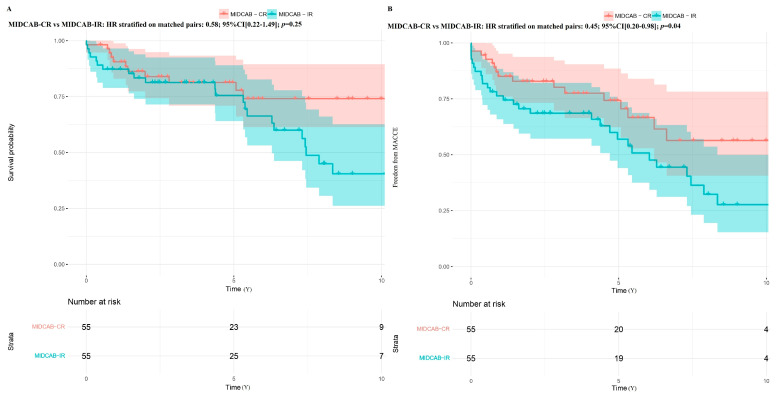
Kaplan–Meier curve probabilities in the matched cohort after minimally invasive direct coronary artery bypass (MIDCAB) with complete revascularization (CR) and incomplete revascularization (IR) strategy. (**A**) Survival probability; (**B**) Freedom from major adverse cardiac and cerebral events (MACCE).

**Figure 8:**
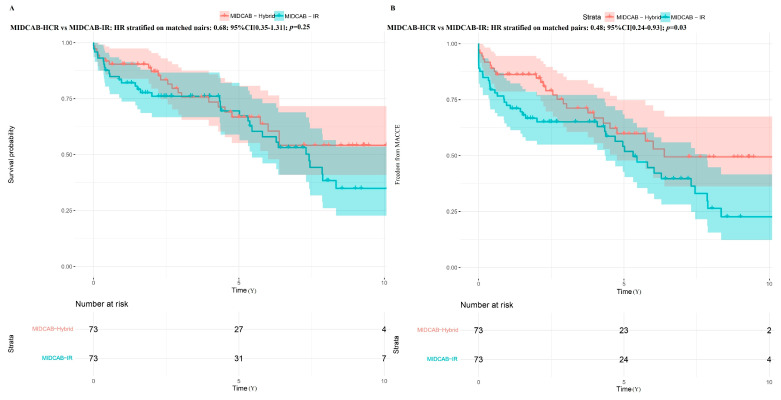
Kaplan–Meier curve probabilities in the matched cohort after minimally invasive direct coronary artery bypass (MIDCAB) with hybrid and incomplete revascularization (IR) strategy. (**A**) Survival probability; (**B**) Freedom from major adverse cardiac and cerebral events (MACCE).

In the propensity-matched populations, patients receiving MIDCAB-CR had comparable mortality (HR stratified on matched pairs: 0.80; 95% CI [0.37–1.79]; *p* = 0.56) and freedom from MACCE (HR stratified on matched pairs: 0.63; 95% CI [0.32–1.24]; *p* = 0.18) compared to patients receiving MIDCAB-HCR (Figure 6). Patients receiving MIDCAB-CR and MIDCAB-HCR had similar survival rates compared to MIDCAB-IR (HR: 0.58; 95% CI [0.22–1.49]; *p* = 0.25) and (HR: 0.68; 95% CI [0.35–1.31]; *p* = 0.25), respectively. However, they experienced a higher freedom from MACCE than patients receiving MIDCAB-IR (HR: 0.45; 95% CI [0.20–0.98]; *p* = 0.04) and (HR: 0.48; 95% CI [0.24–0.93]; *p* = 0.03), respectively (Figure 7 and Figure 8).

## 4. Discussion

This single-center analysis of 480 patients undergoing MIDCAB confirms that this minimally invasive strategy is safe, effective and durable, with a 94.4% LITA–LAD graft patency rate of patients who underwent angiography at follow-up and excellent long-term clinical outcomes in both MIDCAB-CR and MIDCAB-HCR.

The patency of the LITA to LAD graft is the most important factor in predicting long-term outcomes following CABG [13]. The long-term patency rate of the LITA-LAD graft was shown to be excellent, reaching 93% at 15 years [14]. Despite this excellent patency outcome and the advantages of minimally invasive CABG, the adoption rate of this minimally invasive strategy in the surgical community remains low, accounting for less than 1.5% of annual CABG procedures, according to the most recent Society of Thoracic Surgeons database report [1]. The surgical community’s limited adoption of minimally invasive CABG procedures has led to the widespread adoption of PCI [15], even though results comparing MIDCAB LITA to LAD and PCI using second-generation drug-eluting stents favor MIDCAB, which has superior freedom from myocardial infarction and target vessel repeat revascularization [7].

Our early outcomes, including an in-hospital mortality rate of 1.4% and a perioperative myocardial infarction rate of 4.1%, are consistent with the low morbidity and mortality rates reported in large studies, such as the Leipzig cohort. This cohort documented an in-hospital mortality rate of 0.9% across 2667 patients over 22 years [16]. Similarly, Margari et al. [17] reported a 0.8% perioperative mortality rate in a contemporary Italian cohort, and Akintoye et al. [18] observed no significant perioperative mortality over 16 years of follow-up. Recent studies have also highlighted key benefits of MIDCAB, including reduced bleeding, lower transfusion rates, and shorter hospital stays compared to sternotomy CABG [6,19,20]. Since the routine adoption of TTFM in 2022, the incidence of perioperative MI has significantly decreased compared to prior to its routine use (0.57% vs. 6.2%, *p* = 0.018), proving the importance of its intraoperative use during surgery.

Davierwala et al. [16] reported 77.7% survival and greater than 90% LITA-LAD patency after 10 years. Meanwhile, Akintoye et al. [17] observed 81% survival and 96.7% freedom from repeat LAD revascularization after 10 years. Margari et al. [18] reported 87.1% and 84.3% survival at five and 10 years, respectively, confirming the durability of MIDCAB, even in older and more comorbid populations. Repossini et al. [21] reported 79.8% survival and 70.5% freedom from MACCE at 15 years, with 96.8% LITA-LAD patency. Weymann et al. [22] reported 94.3% survival at 10 years following MIDCAB. In our study, the five- and ten-year survival rates were 78% and 60%, respectively, and the freedom from MACCE rates were 66% and 51%, respectively. However, it should be noted that MIDCAB was performed as CR in 54% of patients with single vessel coronary artery disease. In these patients, the five- and ten-year survival rates were 84.6% and 73.5%, respectively, and the freedom-from-MACCE rates were 72.1% and 63.0%, respectively. These rates are in line with those of the aforementioned studies, which primarily concerned patients with single vessel disease.

HCR combining MIDCAB LITA to LAD with PCI to non-LAD areas has been shown to provide results similar to those of full surgical revascularization while minimizing invasiveness [23]. Newman et al. found that “MIDCAB-first” and “PCI-first” approaches in HCR both yielded excellent 10-year survival, with no significant differences in long-term MACCE [24]. Our MIDCAB-HCR group showed similar survival and freedom from MACCE rates compared to MIDCAB-CR group, supporting the idea of a hybrid strategy in multivessel disease where PCI can complement minimally invasive surgery.

Compared to IR, CR is known to be associated with improved long-term outcomes in CABG patients [25,26]. However, CR is not always achievable, and there are certainly cases in which IR is unavoidable, such as in patients with heavily calcified or small target vessels, limited graft material, non-vital myocardium, or major comorbidities. Our subgroup analyses also indicate that MIDCAB-IR is associated with worse survival and MACCE rates. This highlights the importance of complete or hybrid approaches whenever possible.

An observational study certainly has several notable limitations. The main limitation stems from this study’s retrospective nature, which introduces selection bias regarding which patients received HCR or IR. In this cohort, those patients were more comorbid thus associated with higher mortality and MACCE at follow-up. This was partially addressed by PS matching to minimize the impact of potential confounders. However, IR may serve as a surrogate parameter for advanced coronary artery disease and overall comorbidity burden.

## 5. Conclusions

Our single-center experience showed that MIDCAB can be performed safely with excellent long-term survival and freedom from MACCE, especially in patients with isolated proximal LAD disease and in selected high-risk patients with multivessel disease as part of hybrid coronary revascularization. In selected high-risk patients, unsuitable for conventional CABG and HCR, incomplete revascularization via MIDCAB is feasible but with increased incidence of MACCE in the long term.

## Figures and Tables

**Table 1:** Patients’ characteristics.

Factor	MIDCAB	MIDCAB-CR	MIDCAB-HCR	MIDCAB-IR
*n*	*n* = 480	*n* = 260	*n* = 142	*n* = 78
Age (years)	66.57 (9.33)	65.77 (9.50)	66.96 (9.24)	68.58 (8.86)
Age above 75 (%)	86 (19.9)	43 (16.5)	27 (19.0)	16 (20.5)
Female gender (%)	98 (20.4)	58 (22.3)	26 (18.3)	14 (17.9)
BMI (Kg/m^2^)	27.91 (4.49)	27.81 (4.37)	27.69 (4.81)	28.70 (4.27)
Obesity (%)	(26.8)	68 (26.2)	34 (23.9)	27 (34.6)
EF (%)	51.47 (9.58)	53.13 (8.43)	50.18 (10.50)	48.35 (10.43)
EF below 40 (%)	90 (18.7)	33 (12.7)	35 (24.6)	22 (28.2)
Diabetes (%)	151 (31.4)	71 (27.3)	50 (35.2)	30 (38.5)
Insulin (%)	52 (10.8)	19 (7.3)	21 (14.8)	12 (15.4)
Active smoker (%)	86 (17.9)	39 (15.0)	31 (21.8)	16 (20.5)
AF (%)	49 (10.2)	22 (8.5)	15 (10.6)	12 (15.4)
CLD (%)	42 (8.7)	15 (5.8)	16 (11.3)	11 (14.1)
Moderate RI (%)	188 (39.1)	104 (40.0)	56 (39.4)	28 (35.9)
Severe RI (%)	49. (10.2)	19 (7.3)	14 (9.9)	16 (20.5)
Dialysis (%)	6 (1.2)	5 (1.9)	1 (0.7)	0 (0.0)
History of CVAEs (%)	8 (1.6)	3 (1.2)	3 (2.1)	2 (2.6)
History of PCI (%)	131 (27.2)	60 (23.1)	60 (42.3)	11 (14.1)
LM (%)	40 (8.3)	0 (0.0)	19 (13.4)	21 (26.9)
NYHA III IV (%)	32 (6.6)	12 (4.6)	15 (10.6)	5 (6.4)
PAD (%)	82 (17.0)	30 (11.5)	31 (21.8)	21 (26.9)
Recent MI (%)	124 (25.8)	53 (20.4)	49 (34.5)	22 (28.2)
Urgent (%)	171 (35.6)	75 (28.8)	66 (46.5)	30 (38.5)
Euroscore II (%)	1.99 (2.45)	1.75 (2.57)	2.10 (2.01)	2.60 (2.70)

Data-are-expressed-as-mean ± standard-deviation-or-*n* (%). Abbreviations: BMI, body mass index; EF, ejection fraction; AF, atrial fibrillation; CLD, chronic liver disease; RI, renal impairment; CVAEs, cardiovascular adverse events; PCI, percutaneous coronary interventions; LM, left main coronary artery; NYHA, New York Heart Association functional classification; PAD, peripheral artery disease; MI, myocardial infraction. Moderate and severe renal impairment defined as eGFR > 50 < 85 mL/min/1.73 m^2^ and eGFR < 50 mL/min/1.73 m^2^, respectively. CVAEs defined as any history of stroke or transient ischemic attack.

**Table 2:** Early outcomes.

Factor	MIDCAB	MIDCAB-CR	MIDCAB-Hybrid	MIDCAB-IR	*p*	*p*	*p*
*n*	480	260	142	78	CR vs. HCR	CR vs. IR	HCR vs. IR
In hospital MACCE (%)	27 (4.8)	10 (3.8)	7 (4.9)	10 (12.8)	0.60	0.005	0.04
In hospital mortality (%)	7 (1.4)	1 (0.4)	3 (2.1)	3 (3.8)	0.13	0.04	0.45
Postoperative MI (%)	20 (4.1)	9 (3.5)	4 (2.8)	7 (9.0)	0.72	0.05	0.05
Postoperative Stroke (%)	3 (0.6)	0 (0.0)	2 (1.4)	1 (1.3)	0.15	0.15	0.93
MCS (%)	2 (0.4)	0 (0.0)	0 (0.0)	2 (2.6)	>0.99	0.06	0.15
Any PRBC (%)	107 (22.2)	50 (19.2)	37 (26.1)	20 (25.6)	0.11	0.22	0.94
DWI (%)	16 (3.3)	6 (2.3)	4 (2.8)	6 (7.7)	0.75	0.03	0.11
New onset RRT (%)	6 (1.2)	0 (0.0)	2 (1.4)	4 (5.1)	0.15	0.02	0.10
Rethoracotomy (%)	29 (6.0)	16 (6.2)	7 (4.9)	6 (7.7)	0.61	0.62	0.40
LOHS	5.90 (3.85)	5.66 (2.20)	6.03 (4.50)	6.71 (6.39)	0.18	0.03	0.36

Data-are-expressed-as-mean ± standard-deviation-or-*n* (%). Abbreviations: MACCE, major adverse cardiac and cerebrovascular events; MI, myocardial infraction; MCS, mechanical circulatory support; PRBC, packed red blood cells; DWI, deep sternal wound infection; RRT, renal replacement therapy; LOHS, length of hospital stay.

## Data Availability

The data that support the findings of this study are available from the corresponding author, upon reasonable request.

## Supplementary Materials

The following supporting information can be downloaded at: https://www.mdpi.com/article/10.3390/jcm14217590/s1.

## Abbreviations

CABG	Coronary artery bypass grafting
CI	Confidence interval
CR	Complete revascularization
DWI	Deep wound infection
HCR	Hybrid coronary revascularization
HRs	Hazard ratios
IABP	Intra-aortic balloon pump
IQR	Interquartile range
IR	Incomplete revascularization
LAD	Left anterior descending artery
LITA	Left internal thoracic artery
LOHS	Length of hospital stay
MACCE	Major adverse cardiac and cerebral events
MI	Myocardial infarction
MIDCAB	Minimally invasive direct coronary artery bypass grafting
PCI	Percutaneous coronary intervention
pRBC	Packed red blood cell transfusion
PS	Propensity score
RRT	Renal replacement therapy
SD	Standard deviation
SMD	Standardized mean difference
TTFM	Transit time flow measurement
