# Supplementary material for: Long-Term Clinical Outcomes of Minimally Invasive Direct Coronary Artery Bypass Grafting
Source: J Clin Med. 2025 Oct 26;14(21):7590. doi: 10.3390/jcm14217590 (PMC12609691; doi:10.3390/jcm14217590)
Supplement: Supplementary file 1 [file jcm-14-07590-s001.zip › jcm-3840354-supplementary.pdf]

Table representing patients’ characteristics after propensity score matching (CR vs HCR)				
	MIDCAB-CR (n=103)	MIDCAB-HCR (n=103)	P-value	SMD
Age	64.33 (9.49)	66.03 (9.17)	0.193	0.182
Age > 75 (%)	12 (11.7)	16 (15.5)	0.543	0.114
Female gender (%)	25 (24.3)	18 (17.5)	0.304	0.168
BMI	27.46 (4.69)	27.17 (4.01)	0.639	0.066
Obesity (%)	24 (23.3)	21 (20.4)	0.736	0.071
EF	50.89 (9.95)	50.99 (9.64)	0.943	0.010
EF < 40 (%)	23 (22.3)	21 (20.4)	0.865	0.047
Diabetes (%)	31 (30.1)	33 (32.0)	0.880	0.042
Insulin (%)	12 (11.7)	12 (11.7)	>.99	<0.001
Active smoker (%)	22 (21.4)	20 (19.4)	0.863	0.048
AF (%)	8 (7.8)	11 (10.7)	0.631	0.101
CLD (%)	8 (7.8)	10 (9.7)	0.806	0.069
Moderate RI (%)	38 (36.9)	41 (39.8)	0.775	0.060
Severe RI (%)	11 (10.7)	10 (9.7)	>.99	0.032
Dialysis (%)	0 (0.0)	1 (1.0)	>.99	0.140
History of CVAEs (%)	1 (1.0)	1 (1.0)	>.99	<0.001
History of PCI (%)	36 (35.0)	43 (41.7)	0.390	0.140
LM (%)	0 (0)	0 (0)	>.99	<0.001
NYHA.III.IV (%)	8 (7.8)	8 (7.8)	>.99	<0.001
PAD (%)	19 (18.4)	14 (13.6)	0.448	0.133
Recent MI (%)	23 (22.3)	31 (30.1)	0.267	0.177
Urgent (%)	44 (42.7)	40 (38.8)	0.671	0.079
Euroscore II (%)	1.97 (2.46)	1.81 (1.98)	0.599	0.073

Data are expressed as mean±standard deviation or n(%).

Abbreviations: BMI, body mass index; EF, ejection fraction; AF, atrial fibrillation; CLD, chronic liver disease; RI, renal impairment; CVAEs, cardiovascular adverse events; PCI, percutaneous coronary interventions; LM, left main coronary artery; NYHA, New York Heart Association functional classification; PAD, peripheral artery disease; MI, myocardial infraction

Table representing patients' characteristics after propensity score matching (CR vs IR)				
	MIDCAB-CR (n=55)	MIDCAB-IR (n=55)	P-value	SMD
Age	65.04 (11.18)	67.60 (8.97)	0.187	0.253
Age > 75 (%)	12 (21.8)	10 (18.2)	0.812	0.091
Female gender (%)	11 (20.0)	10 (18.2)	>.99	0.046
BMI	28.38 (5.31)	29.42 (4.05)	0.251	0.220
Obesity (%)	19 (34.5)	22 (40.0)	0.694	0.113
EF	50.51 (10.31)	49.96 (9.69)	0.776	0.055
EF < 40 (%)	15 (27.3)	12 (21.8)	0.658	0.127
Diabetes (%)	25 (45.5)	22 (40.0)	0.700	0.110
Insulin (%)	9 (16.4)	9 (16.4)	>.99	<0.001
Active smoker (%)	12 (21.8)	11 (20.0)	>.99	0.045
AF (%)	4 (7.3)	6 (10.9)	0.742	0.127
CLD (%)	8 (14.5)	7 (12.7)	1.000	0.053
Moderate RI (%)	23 (41.8)	19 (34.5)	0.556	0.150
Severe RI (%)	6 (10.9)	10 (18.2)	0.418	0.207
Dialysis (%)	0 (0)	0 (0)	>.99	<0.001
History of CVAEs (%)	1 (1.8)	1 (1.8)	>.99	<0.001
History of PCI (%)	10 (18.2)	7 (12.7)	0.599	0.151
LM (%)	0 (0)	0 (0)	>.99	<0.001
NYHA.III.IV (%)	3 (5.5)	3 (5.5)	>.99	<0.001
PAD (%)	15 (27.3)	13 (23.6)	0.827	0.084
Recent MI (%)	11 (20.0)	12 (21.8)	>.99	0.045
Urgent (%)	16 (29.1)	18 (32.7)	0.837	0.079
Euroscore II (%)	1.91 (1.88)	2.29 (2.69)	0.391	0.165

Data are expressed as mean±standard deviation or n(%).

Abbreviations: BMI, body mass index; EF, ejection fraction; AF, atrial fibrillation; CLD, chronic liver disease; RI, renal impairment; CVAEs, cardiovascular adverse events; PCI, percutaneous coronary interventions; LM, left main coronary artery; NYHA, New York Heart Association functional classification; PAD, peripheral artery disease; MI, myocardial infraction

Table representing patients' characteristics after propensity score matching (HCR vs IR)

	MIDCAB-HCR (n=73)	MIDCAB-IR (n=73)	P-value	SMD
Age	68.26 (8.65)	68.26 (8.92)	>.99	<0.001
Age > 75 (%)	17 (23.3)	15 (20.5)	0.842	0.066
Female gender (%)	13 (17.8)	13 (17.8)	>.99	<0.001
BMI	28.94 (5.40)	28.67 (4.34)	0.744	0.054
Obesity (%)	30 (41.1)	25 (34.2)	0.495	0.142
EF	51.23 (10.44)	48.75 (10.40)	0.153	0.238
EF < 40 (%)	16 (21.9)	19 (26.0)	0.699	0.096
Diabetes (%)	25 (34.2)	30 (41.1)	0.495	0.142
Insulin (%)	13 (17.8)	12 (16.4)	>.99	0.036
Active smoker (%)	13 (17.8)	16 (21.9)	0.679	0.103
AF (%)	8 (11.0)	10 (13.7)	0.802	0.083
CLD (%)	8 (11.0)	9 (12.3)	>.99	0.043
Moderate RI (%)	29 (39.7)	27 (37.0)	0.865	0.056
Severe RI (%)	10 (13.7)	14 (19.2)	0.504	0.148
Dialysis (%)	0 (0)	0 (0)	>.99	<0.001
History of CVAEs (%)	1 (1.4)	2 (2.7)	>.99	0.097
History of PCI (%)	10 (13.6)	10 (13.6)	>.99	<0.001
LM (%)	15 (20.5)	15 (20.5)	>.99	<0.001
NYHA.III.IV (%)	8 (11.0)	5 (6.8)	0.563	0.145
PAD (%)	16 (21.9)	19 (26.0)	0.699	0.096
Recent MI (%)	26 (35.6)	19 (26.0)	0.282	0.209
Urgent (%)	37 (50.7)	28 (38.4)	0.183	0.250
Euroscore II (%)	2.34 (2.45)	2.40 (2.27)	0.871	0.027

Data are expressed as mean±standard deviation or n(%).

Abbreviations: BMI, body mass index; EF, ejection fraction; AF, atrial fibrillation; CLD, chronic liver disease; RI, renal impairment; CVAEs, cardiovascular adverse events; PCI, percutaneous coronary interventions; LM, left main coronary artery; NYHA, New York Heart Association functional classification; PAD, peripheral artery disease; MI, myocardial infraction
